# Influence of *Agaricus bisporus* Mushroom on Pb Toxicokinetic in Pregnant Rats

**DOI:** 10.3390/ijerph20043114

**Published:** 2023-02-10

**Authors:** Érika Leão Ajala Caetano, Tatiana Pedron, Bruna Moreira Freire, Camila Neves Lange, Bruno Lemos Batista, Denise Grotto

**Affiliations:** 1 Toxicological Research Laboratory–Lapetox, University of Sorocaba, Sorocaba 18023-000, SP, Brazil; 2 Center for Natural and Human Sciences, Federal University of ABC, Santo André 09210-580, SP, Brazil; 3 CIQ-UP Department of Chemistry and Biochemistry, Faculty of Sciences, University of Porto, 4099-002 Porto, Portugal

**Keywords:** lead, *Agaricus bisporus*, combined exposure, toxicokinetic, pregnancy

## Abstract

(Pb) is a toxic metal, responsible for several damages to human health. *Agaricus bisporus* (Ab) is a mushroom with promising antioxidant properties to be used as an alternative chelator in Pb intoxication. The aim was to understand the Pb toxicokinetic and the potential of Ab as a protective agent. A total of 20 female Wistar rats were distributed into 4 groups (n = 5/group): Control (receiving water); Group Ab 100 mg/kg (gavage); Group Pb 100 mg/L in water; and Group Ab + Pb—100 mg/kg + 100 mg/L (gavage and water). Pb administration occurred daily until the 19th day of pregnancy. On day 19 of gestation, the rats were euthanized, and the blood and tissues were collected for Pb measurement, using an inductively coupled plasma mass spectrometer. The results showed that the levels of Pb in the blood, placenta, and liver of the mothers, and in the brain of the fetuses increased significantly in the Pb group. On the other hand, the combined exposure to Pb + Ab showed a significant decrease in the metal concentration in relation to the Pb group, returning to normal levels. Kidney and bone lead levels also increased significantly in the Pb group. However, in the combined exposure group, levels did not return to the control amounts; there was protection, but the Pb concentration was still significantly higher than in the control. In the brain, no significant differences were observed. In conclusion, we suggest *A. bisporus* is a natural chelator, because the co-administration of the mushroom was able to interact with Pb ions, minimizing the Pb absorption and distribution. These effects are suggested since *A. bisporus* have antioxidants and beta glucan that interact with Pb, chelating it and, thus, reducing its toxic effects.

## 1. Introduction

Lead (Pb) is a non-essential element with a proven neurotoxic effect, and with a significant risk for humans exposed to it by environmental, occupational, or dietary factors [1]. Sensitivity to Pb is greater in children; therefore, pregnant women should be aware of the risk of exposure to Pb, because the metal can pass to the fetus during pregnancy through the placenta, and breastfeeding can also be another source of exposure for babies [2,3].

In the general population, gastrointestinal absorption is the most common route, and adults can absorb a maximum of 10% of ingested Pb, in contrast to children (between 2 months and 6 years of age), who can absorb up to 50% of the amount ingested. Considering the toxic effects, in adults, the most common damages are cardiovascular, hypertension, loss of appetite, hallucinations, headache, insomnia, and joint pain [4], while children are susceptible to neurological damage, delay in psychomotor development, and learning difficulties, among others [5,6]. Another well-known toxic effect is the inhibition of the body’s ability to produce hemoglobin, affecting the synthesis of the heme group, by inhibiting the activity in the enzymes, δ-aminolevulinic acid dehydratase (ALA-D), coproporphyrinogen oxidase, and ferrochelatase. Such inhibition causes mild to moderate anemia in adults and severe anemia in children [7]. From the blood, Pb is distributed to all tissues (especially soft tissues such as the kidneys and liver), including the fetus during pregnancy. Subsequently, about 90% of the Pb is deposited in bones [8,9].

The World Health Organization’s 2021 update on the impact of chemicals on public health determined that, of the 2 million people who died in 2019 from exposure to known chemicals, nearly half were due to Pb exposure. Pb exposure is estimated to be responsible for 21.7 million years lost to disability and death [10]. Regarding blood Pb reference values, the Centers for Disease Control and Prevention (CDC) uses a reference value of 3.5 μg/dL to identify children with high Pb levels. The CDC estimates that approximately 800 million children worldwide have Pb in their blood [3]. In addition to affecting adults and children, the release of Pb into the environment can cause mortality and also limit population size among non-human animals, such as populations of wild animals and waterfowl species [11].

The administration of a chelating agent is the current therapy for metal intoxication [12]. EDTACaNa2 (disodium calcium ethylenediaminetetracetic acid), BAL (Dimercaprol), and DMSA (dimercaptosuccinic acid) are some of the synthetic chelating agents used to remove Pb from the body. However, some adverse reactions have already been reported during treatment, such as gastrointestinal discomfort, skin reactions, and elevation of liver enzymes, in addition to the chelation of essential metals [13,14]. In this way, alternative therapies using natural substances such as garlic, vitamin C, and vitamin E have been studied. Some findings have shown the use of different nutrients; for example, vitamins, flavonoids, and mineral elements as defenders against Pb poisoning [15,16,17].

Garlic, a natural substance used in food, contains several essential nutrients and antioxidants, as well as being rich in flavonoids and selenium [18]. It is recommended as an active antioxidant to combat toxicity caused by heavy metals, and the natural chelating ability of the allicin and sulfhydryl groups present in garlic makes it a strong antioxidant in the treatment of lead-induced toxicity, especially long-term toxicity [17,19]. Vitamin E acts as an antioxidant, inhibiting lipid peroxidation. Vitamin E neutralizes the oxidative stress generated by Pb, inhibiting the additional production of free radicals by chelation [17,20].

In this way, culinary and medicinal mushrooms can also be suggested as a complementary therapy. The mushroom *Agaricus bisporus* is highly nutritious [21]. This mushroom contains many bioactive compounds, including alkaloids, flavonoids, steroids, terpenes, and phenolic compounds responsible for reducing oxidative damage [22,23]. In addition, *A. bisporus* also has glucans, fibers, and these polysaccharides, especially the triple helix 1,3-1,6-β-d-glucans, which showed chelating characteristics [24], along with chitin and chitosan, which are known for their antioxidant activity, have the ability to eliminate different species of oxygen radicals, such as alkyl, superoxide, and hydroxyl. The mechanism is still unclear but must be related to the chelation of free metal ions by hydroxyl and amino polysaccharide groups, which leads to the formation of a stable system [25]. In waste treatment, chitosan is investigated as a heavy metal chelator [25,26].

In this way, we evaluated the presence of Pb in the blood and tissues of pregnant rats exposed to the metal, and in the brains of their fetuses, aiming to understand the Pb toxicokinetic in the presence of *A. bisporus* as a protective agent.

## 2. Materials and Methods

Fresh *A. bisporus* mushrooms were obtained commercially from a local producer. The mushrooms were sliced and dried in a ventilated oven at 38 ± 2 °C, until constant mass. The dried mushroom was ground in a mill to obtain a kind of thin flour. The mushroom flour was given to the animals every day as described below.

### 2.1. Experimental Design

Healthy Wistar rats, male and female, were obtained from the UNICAMP vivarium, Campinas, São Paulo state. The experiment was approved by the Committee for the Care and Use of Experimental Animals of the University of Sorocaba, under the protocol number 175/2020. The research was developed in the experimental animal vivarium at the Laboratory of Toxicological Research-LAPETOX, University of Sorocaba. The procedures followed the ethical precepts and the animal welfare standards [27]. Rats were kept in boxes with constant cycles of light and dark cycles—12 h each—at a temperature of 21–23 °C, with free access to a standard chow diet and filtered water. The animals were kept for a week in adaptation before starting the procedures.

For the induction of pregnancy, 01 male and 03 females were mated at night in an independent box, in the proestrus and estrus phases of the estrous cycle. Day 1 of gestation occurred when sperm were found in the vaginal wash, performed in the morning after mating [28].

All pregnant rats were randomly divided into 4 groups (n = 5 per group): Group I—Control (received water); Group II—Ab 100 mg/kg (received *A. bisporus* mushroom); Group III—Pb 100 mg/L (received Pb); Group IV—Ab + Pb (100 mg/kg +100 mg/L; combined exposure). Exposure to Pb was made in drinking water, while Ab was solubilized in water and administered orally (by gavage). The administration of Pb and Ab was carried out until the 19th day.

The dose of 100 mg/kg/day of *A. bisporus* was defined based on previous studies from our team [29,30]. The Pb dose of 100 mg/L was defined in a pilot study, which found out that the dose represented values above 90 μg/dL in the blood of pregnant rats. These levels can cross the placental barrier, causing damage to the cognitive development of the fetus [31,32]. At the end of the experiment, the animals were euthanized with ketamine, xylazine, and acepromazine. Blood and tissues were collected for Pb measurement.

### 2.2. Determination of Pb Levels

Pb was quantified using an inductively coupled plasma mass spectrometer, and the operating conditions are reported in Table 1. Reference materials (blood and tissue) were used as a control of the quality of the analysis. Recovery of blood reference material (Seronorm Trace Elements Blood L-2, ALS Scandinavia AB, Lulea, Sweden) was (103%). Concerning tissue (CRL-ISS 12th Proficiency Test Bovine meat), the recovery was (105.48%).

For blood and tissue, the methodological limits of detection were 1.9535 µg/L and 0.0435 µg/L, respectively. The methodology of Batista and colleagues [33] with some modifications was used for the determination of Pb in blood. In metal-free tubes, 100 µL of blood was added with 4.9 mL of a solution containing 0.5% v v^−1^ of HNO_3_ and 0.01% v v^−1^ of Triton X-100. The samples of blood were shaken and analyzed by ICP-MS.

Tissues were lyophilized for 48 h (lyophilizer Liotop l101, Liobras, São Carlos, SP, Brazil). After that, 35–250 mg of the dried sample was added in metal-free tubes and pre-digested for 48 h with 2 mL of sub-distilled HNO_3_. The samples were then heated at 90 °C, for 4 h. After cooling, the volume was made up to 50 mL with ultrapure water for analysis by ICP-MS [34], with modifications.

### 2.3. Statistical Analysis

Data were reported as mean ± standard deviation (SD). The comparison of the mean values among the experimental groups were determined by one-way nonparametric ANOVA (Kruskal–Wallis test), followed by Tukey–Kramer’s interval tests. Differences were considered statistically significant when *p* < 0.05. The results were analyzed using the Statistica^®^ 8.0 and Graph Pad Prism^®^ 5 programs.

## 3. Results

**Figure 1:**
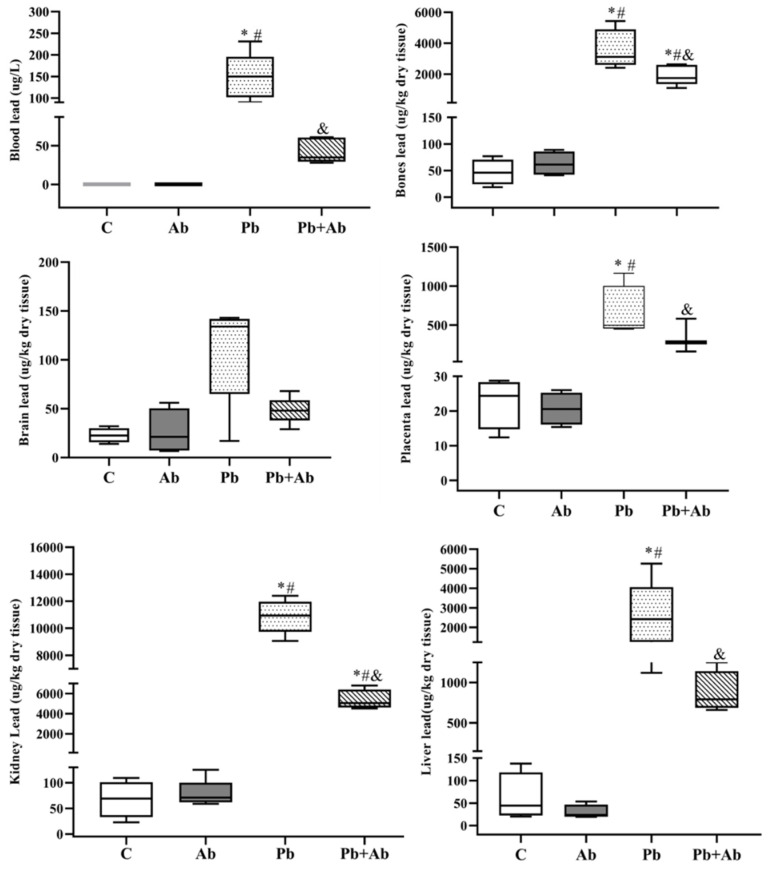
Lead concentrations (mean ± standard deviation) in blood and tissue samples from pregnant rats exposed to *A. bisporus* and lead, from gestation day 1 to 19. C (Control), Ab (*A. bisporus* 100 mg/kg/day), Pb (Lead 100 mg/L/day), Pb + Ab (100 mg/L/day of Pb + 100 mg/kg/day of *A. bisporus*), n = 5/group. * *p* < 0.05 in comparison to Control group, # in comparison to *A. bisporus*, and & in comparison to lead. Kruskal–Wallis test, followed by Tukey–Kramer’s.

The Pb concentrations in the pregnant rats in the blood, bone, brain, placenta, liver, and kidney samples are shown in Figure 1 and in the Supplementary Material.

A significant increase in the Pb concentration was observed in the blood, placenta, and liver in the Pb group. On the other hand, the group that received both Pb + Ab showed a significant drop in the metal concentration in relation to the Pb group. The blood, brain, placenta, and liver did not present statistical differences in lead levels when comparing the control and Pb + Ab. Numerically, it seems to be a difference; however, the large variation within each group (see Supplementary Material) may have been responsible for the lack of significance. In the placenta, a tendency was found when comparing the Pb + Ab group to the control (*p* = 0.62). The lead concentration in the kidneys and bones also increased significantly in the Pb group. However, in the Pb + Ab group, despite the levels did not return to the similarity of the control, the lead levels diminished compared to the Pb group.

**Figure 2:**
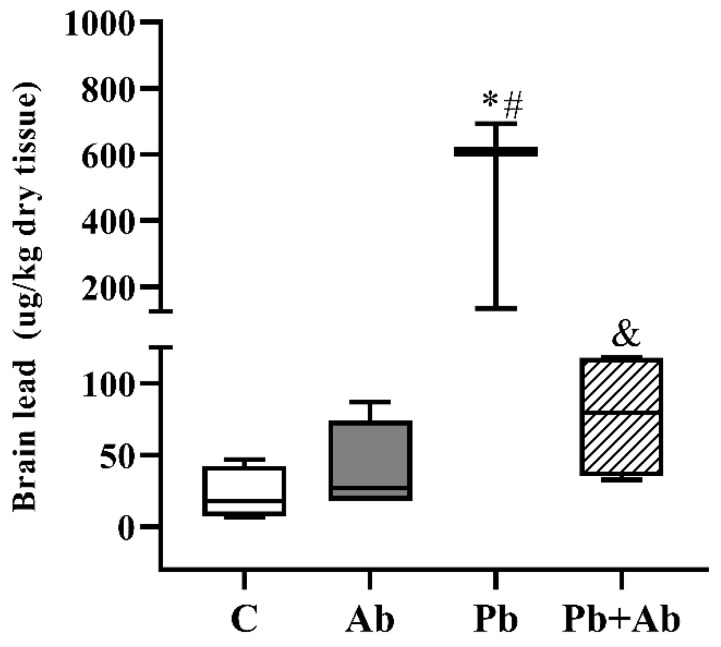
Lead concentrations (mean ± standard deviation) in brain samples of fetuses from female rats exposed to *A. bisporus* and lead, from gestation day 1 to 19. C (Control), Ab (*A. bisporus* 100 mg/kg/day), Pb (Lead 100 mg/L/day), Pb + Ab (100 mg/L/day of Pb + 100 mg/kg/day of *A. bisporus*), n = 5/group. * *p* < 0.05 in comparison to Control group, # in comparison to *A. bisporus*, and & in comparison to lead. One-way ANOVA, followed by the Tukey–Kramer’s test.

Concerning the brain of the fetuses, the Pb levels are shown in Figure 2. A significant increase in the Pb concentration was observed in the Pb group, compared to the control. On the other hand, the Pb + Ab group showed a significant drop in the metal levels in relation to the Pb group, showing the mushroom has a chelator potential.

## 4. Discussion

Nowadays, Pb toxicity is still a key environmental health problem causing harmful effects for children and pregnant women, but not restricted to them. Several and distinct species of wild animals are affected by Pb poisoning in the environment [11]. Pb toxic effects on pregnancy are complex, and Pb can affect the mother and fetus with harmful effects and prolonged consequences [35].

The concentrations of Pb in the blood reflect the amount that enters the body through all exposure routes through the respiratory and gastrointestinal tracts, and skin [36]. Thus, we quantified the Pb concentrations in the blood of pregnant rats to assess their level of exposure. The mean blood Pb level for the pregnant rats in the Pb group was around 140 μg/L. This value is two times greater than the maximum allowed biological index—60 µg/L—for Pb in the blood, in Brazil [37]. This finding was similar to those reported by Saleh et al. (2018) [38], with pregnant rats exposed to 160 and 320 mg Pb/kg for 20 days. Those authors found blood lead concentrations of 100 and 125 μg/dL, respectively, and these concentrations are clinically toxic.

We positively observed a decrease in the level of Pb in maternal blood with the *A. bisporus* co-exposures (around 50 μg/dL, almost three times lower than the Pb group) being below the maximum permitted biological index. Comparatively, similar data were reported by Sadeghi et al. (2021) [39], in which pregnant rats were exposed to Pb and treated with vitamin C and garlic extract. These findings corroborated with several studies and indicated that antioxidants can reduce toxic metals in the blood [40,41].

In the current study, a significant rise in the Pb level was observed in the kidney and hepatic tissues of the Pb-exposed group. This increase was in accordance with results reported by Takano et al. (2015) [42], for animals exposed to Pb for 4 weeks. The high concentrations of Pb in the maternal liver were probably caused by an altered Pb pharmacokinetics in pregnancy. Since the kidney was identified as a soft tissue target for Pb accumulation, research has been published on the Pb concentration in animal kidneys [43,44]. One possible explanation is that the Pb distribution is due to its reabsorption. In the kidney, Pb is filtered in the glomerulus; however, most of the Pb filtered is reabsorbed by the distal tubule and collecting duct [45].

During pregnancy, the internal store of Pb can escape from the bone, due to high bone turnover, and cross the placenta by diffusion in the developing embryo [46]; in our study, we found high concentrations of bone lead in the group exposed to Pb (3621.20 ± 1249.23). However, in the Pb + Ab group (1932.40 ± 657.63), the concentrations did not return to the similarity of the control group. This finding is in agreement with that reported by Mumtaz et al. (2020) [6], who reported that bones are one of the main storage sites for Pb, with the bone matrix being the main target due to its ability to replace cations (Fe^2+^, Ca^2+^, and Mg^2+^ Na^+^) in the body, altering the mineral metabolism.

The accumulation of Pb in the body of women of reproductive age can be transferred to the fetus during pregnancy through the placenta, the structure responsible for fetal nutrition, allowing the passage of Pb to the fetus, a fact that increases the risk of pregnancy complications and appearance of diseases after birth [47]. Thus, concentrations of Pb in the placenta can be good biomarkers of fetal exposure to Pb [48]. In our study, we found high Pb concentrations in the placenta of the group exposed to Pb (around 652 µg/L). On the other hand, *A. bisporus* co-exposure decreased the Pb concentration by half (about 340 µg/L), suggesting that the co-administration of the mushroom *A. bisporus* was beneficial.

In fetuses, the blood–brain barrier, because it is not fully developed, becomes the gateway to toxic products. For this reason, studies suggest that Pb can enter the brain more easily, becoming more harmful to newborns than adults [49,50]. In our study, an increase in the Pb concentrations in the brain of the fetuses in the group exposed to Pb (478.33 ± 301.21) was found, unlike in the Pb + Ab group where we observed a decrease in the metal (77.61 ± 46.42), returning to normal concentrations. In a study carried out by Antonio-Garcia and Masso-Gonzalez (2008) [51], rats were exposed to 300 mg Pb/L, a higher dose than in our study, and to a natural antioxidant, during pregnancy and lactation. The results were similar to ours, with an increase in Pb levels in the brain of the fetuses, showing an alteration in the antioxidant defense systems, as well as a reduction in the Pb concentrations in the groups exposed to antioxidants such as vitamin C. Thus, we can hypothesize that the exogenous supplementation of antioxidant agents, such as the mushroom *A. bisporus*, may be an alternative therapy for Pb toxicity due to its ability to cross the blood–brain barrier and exert its inhibitory effects on the brain. In studies carried out by Pachauri et al. (2008) [52] and Flora et al. (2003) [53], both highlighted the importance of antioxidant supplementation in metal poisoning, as supplementation can be beneficial to increase metal mobilization. In addition to the mushroom having antioxidants, it has important amounts of beta glucan, a soluble fiber that is directly related to the Pb metabolism. According to Visweswar et al. (2008) [54], three cyclic beta glucans interact with Pb, chelating it and, thus, reducing its toxic effects.

## 5. Conclusions

In this study, we observed that Pb has a rapid distribution for both the mother and fetus. Therefore, we conclude that there is a link between the Pb concentrations in the blood and tissues of the mothers and in the pups’ brain, demonstrating Pb transfer across the placenta. In addition, co-administration of the mushroom *A. bisporus* was able to interact with Pb ions. These results highlight the potential use of *A. bisporus* as neuroprotective agents from Pb-induced harmful effects.

## Figures and Tables

**Table 1:** Operating conditions for ICP-MS.

ICP-MS: Operating Conditions	
Monitored isotope	208Pb
Radio frequency power	1550 W
Argon flow	15 L min^−1^
Nebulization chamber	0.9 L min^−1^
Collision cell	Helium (purity > 99.999%)
Nebulization chamber	Scott (double pass)
Interface	Nickel cones
Sample	0.90 mm
Skimmer	0.45 mm

## Data Availability

Data and publication materials are available from the corresponding author upon a reasonable request.

## Supplementary Materials

The following supporting information can be downloaded at: https://www.mdpi.com/article/10.3390/ijerph20043114/s1, Table S1: Lead levels in different tissues.
