# Supplementary material for: Influence of Agaricus bisporus Mushroom on Pb Toxicokinetic in Pregnant Rats
Source: Int J Environ Res Public Health. 2023 Feb 10;20(4):3114. doi: 10.3390/ijerph20043114 (PMC9965185; doi:10.3390/ijerph20043114)
Supplement: Supplementary file 1 [file ijerph-20-03114-s001.zip › ijerph-2199841-supplementary.pdf]

Supplementary Material Table S1. Lead levels in different tissues.

Parameter	Groups			
	C	Ab	Pb	Pb+Ab
**LIVER**				
Mean ± Standard deviation	62 ± 53	31 ± 15	2,614 ± 1,644	716 ± 362
Minimum and Maximum	20 - 138	19 – 54	1,119 – 5,264	196 – 1,248
**PLACENTA**				
Mean ± Standard deviation	22 ± 7	21 ± 5	704 ± 402	531 ± 406
Minimum and Maximum	12 - 29	15 - 26	448 – 1,167	166 – 1,082
**KIDNEY**				
Mean ± Standard deviation	68 ± 36	79 ± 27	10,868 ± 1,251	5,359 ± 1,002
Minimum and Maximum	23 - 109	59 - 125	9,061 – 12,407	4,524 – 6,802
**BONES**				
Mean ± Standard deviation	47 ± 24	63 ± 23	3,621 ± 1,249	1,932 ± 657
Minimum and Maximum	19 - 77	41 - 88	2,412 – 5,436	1,106 – 2,634
**BRAIN**				
Mean ± Standard deviation	23 ± 7	26 ± 23	109 ± 53	48 ± 14
Minimum and Maximum	14 - 32	7 - 57	17 - 143	29 - 68
